# OncomiRs miR-106a and miR-17 negatively regulate the nucleoside-derived drug transporter hCNT1

**DOI:** 10.1007/s00018-021-03959-8

**Published:** 2021-10-13

**Authors:** Clara Boces-Pascual, Aida Mata-Ventosa, Mireia Martín-Satué, Loreto Boix, Meritxell Gironella, Marçal Pastor-Anglada, Sandra Pérez-Torras

**Affiliations:** 1 Molecular Pharmacology and Experimental Therapeutics, Department of Biochemistry and Molecular Biomedicine, Institute of Biomedicine, University of Barcelona (IBUB), Barcelona, Spain; 2 Centro de Investigación Biomédica en Red de Enfermedades Hepáticas y Digestivas (CIBER EHD), Instituto de Salud Carlos III, Madrid, Spain; 3 Institut de Recerca Sant Joan de Déu (IR SJD-CERCA), Esplugues de Llobregat, Barcelona, Spain; 4 Department of Pathology and Experimental Therapeutics, Faculty of Medicine and Health Sciences, Campus of Bellvitge, University of Barcelona, Hospitalet de Llobregat, Barcelona, Spain; 5 Biomedical Research Institute of Bellvitge (IDIBELL), Oncobell Program, L’Hospitalet de Llobregat, Barcelona, Spain; 6 Centro de Investigación Biomédica en Red Cáncer (CIBERONC), Instituto de Salud Carlos III, Madrid, Spain; 7 Barcelona Clinic Liver Cancer (BCLC) Group, Liver Unit, Hospital Clínic of Barcelona, Institut d’Investigacions Biomèdiques August Pi i Sunyer (IDIBAPS), Fundació Clínic per a la Recerca Biomèdica (FCRB), University of Barcelona, Barcelona, Spain; 8 Gastrointestinal & Pancreatic Oncology Group, Hospital Clinic of Barcelona/Institut d’Investigacions Biomèdiques August Pi i Sunyer (IDIBAPS), Barcelona, Spain

**Keywords:** Non-coding RNA, Nucleoside analog, Nucleoside transporter, CNT1, Chemoresistance

## Abstract

High-affinity uptake of natural nucleosides as well as nucleoside derivatives used in anticancer therapies is mediated by human concentrative nucleoside transporters (hCNTs). hCNT1, the hCNT family member that specifically transports pyrimidines, is also a transceptor involved in tumor progression. In particular, oncogenesis appears to be associated with hCNT1 downregulation in some cancers, although the underlying mechanisms are largely unknown. Here, we sought to address changes in colorectal and pancreatic ductal adenocarcinoma—both of which are important digestive cancers—in the context of treatment with fluoropyrimidine derivatives. An analysis of cancer samples and matching non-tumoral adjacent tissues revealed downregulation of hCNT1 protein in both types of tumor. Further exploration of the putative regulation of hCNT1 by microRNAs (miRNAs), which are highly deregulated in these cancers, revealed a direct relationship between the oncomiRs miR-106a and miR-17 and the loss of hCNT1. Collectively, our findings provide the first demonstration that hCNT1 inhibition by these oncomiRs could contribute to chemoresistance to fluoropyrimidine-based treatments in colorectal and pancreatic cancer.

**Graphic abstract:**

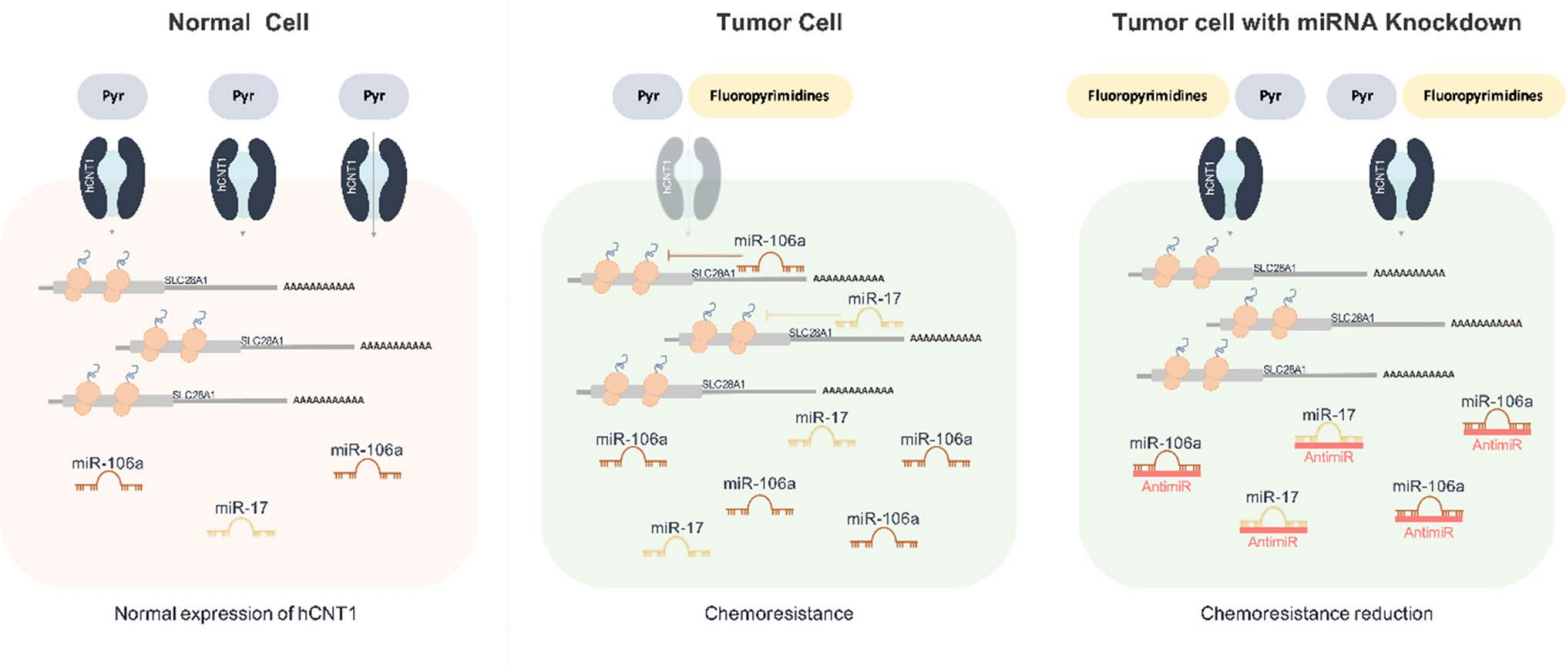

**Supplementary Information:**

The online version contains supplementary material available at 10.1007/s00018-021-03959-8.

## Introduction

MicroRNAs (miRNAs) are small non-coding RNAs 18–25 nucleotides in length that negatively regulate the translation and/or stability of target transcripts [1]. miRNAs control the expression of a large number of transcripts either through direct binding or by targeting transcripts encoding transcription factors, epigenetic regulators, or effectors of signal transduction pathways [2]. As a class, miRNAs are major regulators of cell function and homeostasis that are aberrantly expressed in many different pathologies, including inflammatory diseases and cancers. Deregulated miRNAs have a profound impact on cancer, mainly owing to their role in regulating the expression of genes involved in various hallmarks of cancer, including proliferation, invasion, apoptosis, and angiogenesis [3]. Alterations include upregulation of oncogenic miRNAs (oncomiRs) and downregulation of miRNAs that negatively regulate oncogenes [4]. Moreover, miRNA signatures make it possible to discriminate between healthy and cancer tissues and among different subtypes of a given type of cancer [5]. The role of miRNAs in the development of chemoresistance is well established in different cancers and has been shown to involve modulation of more than a single specific target [6]. In fact, modulating the levels of one individual miRNA can simultaneously affect many complex molecular pathways [7].

Nucleoside analogs have often been used in the treatment of solid tumors and lymphoproliferative malignancies. Nucleoside transporters (NTs) are responsible for the cellular uptake of nucleosides and nucleoside analogs used to treat cancer and are the first limiting step in controlling the bioavailability of these drugs. NTs are encoded by two different solute carrier (SLC) gene families, *SLC28* and *SLC29*, that differ in their substrate selectivity, affinity, and location. *SLC29* genes encode the four members of the human equilibrative nucleoside transporter (hENT) family. hENT1 and hENT2 are the family members responsible for the uptake of purines and pyrimidines at the cell membrane. Their affinity is on the higher side of the micromolar range, and they are ubiquitously expressed. *SLC28* genes encode the three members of human concentrative nucleoside transporter (hCNT) family, which show substrate affinity constants in the low micromolar range. Although these three hCNT members transport uridine, they differ in their substrate selectivity. hCNT1 mediates the uptake of pyrimidines and hCNT2 mediates the uptake of purines, whereas hCNT3 can transport both purines and pyrimidines. The affinity and selectivity for nucleoside analog drugs are also different among these isoforms, resulting in different efficacies of purine and pyrimidine analogs in treating different types of cancers. In general, purine derivatives show efficacy against hematological malignancies, whereas pyrimidine analogs are also effective against solid tumors [8]. hCNT proteins were originally detected only in polarized epithelia, but subsequent work has extended their expression range. In polarized epithelia, hCNT proteins are preferentially located at the apical side, enabling vectorial flux to be mediated in a coordinated manner with hENT proteins present at the basolateral side [9–11].

Several recent studies have challenged the focus on hCNTs as mere substrate translocators. An analysis of the enteric transportome in Crohn’s disease showed a significant decrease in the pyrimidine transporter hCNT1 and the purine transporter hCNT2 [12]. In particular, additional transceptor properties of hCNT1 relevant to tumor progression have reinforced evidence of a role for this transporter in regulating cell physiology beyond its canonical function as a nucleoside translocator [13]. Notably in this context, oncogenesis is often associated with downregulation of hCNT1 in different tumors [14–19]. Nevertheless, the nature of changes in hCNT1 that occur in important digestive cancers, such as colorectal cancer (CRC) and pancreatic ductal adenocarcinoma (PDAC), and how this downregulation contributes to carcinogenesis, is largely unknown. Thus, considering the relevant functions of hCNT1 as a drug transporter and taking into account the considerable deregulation of miRNAs in these cancers, we sought to address the potential regulation of hCNT1 by miRNAs in colorectal and pancreatic cancers. Our findings demonstrate that the oncomiRs, miR-106a and miR-17, regulate expression of the nucleoside transporter hCNT1 in colorectal and pancreatic cancer and thereby contribute to chemoresistance to fluoropyrimidine-based treatments.

## Materials and methods

### Clinical samples

**Fig. 1:**
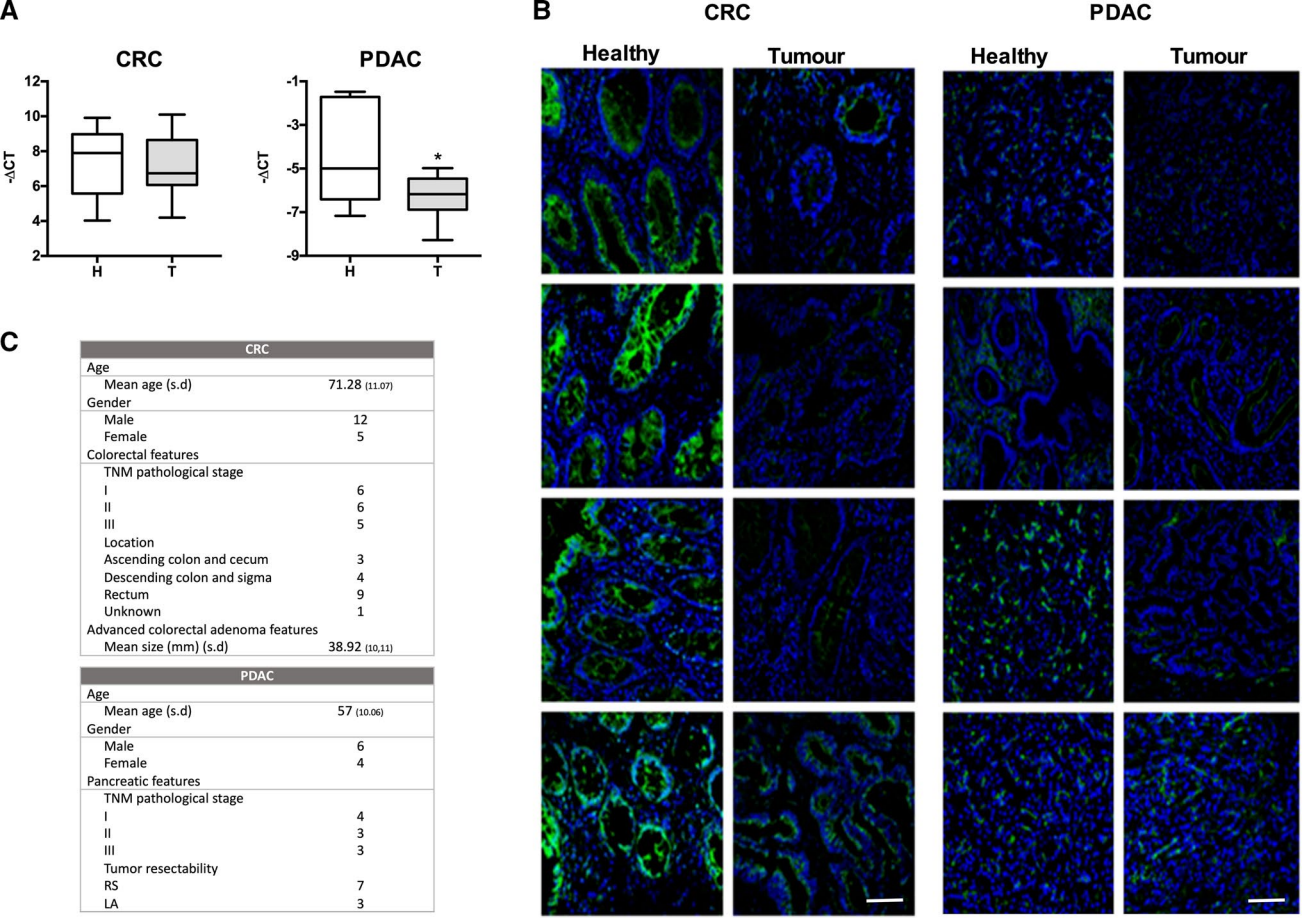
hCNT1 is decreased in tumors vs healthy adjacent tissue in colorectal cancer (CRC) and pancreatic ductal adenocarcinoma (PDAC). **A** hCNT1 mRNA levels in CRC (*n* = 17) or PDAC (*n* = 9) clinical samples, comparing tumor and normal adjacent tissue. Statistical significance was determined with Student’s *t* test. **B** Immunofluorescence images of hCNT1 protein (green) in four, paired, independent clinical samples of tumor and normal adjacent CRC and PDAC tissue. hCNT1 was detected using a polyclonal anti-CNT1 antibody. All the images were obtained under the same magnification. Scale bars, 75 µm. **C** Patient and tumor samples characteristics, including the number of samples in each category. s.d., standard deviation; RS, resectable; LA, locally advanced

Clinical samples were obtained from the Biobank facilities of IDIBAPS-Hospital Clinic of Barcelona. Tumoral and adjacent non-tumoral tissues, including 17 for CRC (12 paired), 22 for HCC (all of them paired) and 10 for PDAC (6 paired), were obtained from patients after surgical resection in accordance with the institutional policy. None of the patients had received chemo- or radiotherapy before sample collection. The clinico-pathological features of all individuals included in the study are detailed in Fig. 1C. Resected cancer tissues were immediately cut and frozen in liquid nitrogen and stored at − 80 °C or embedded in O.C.T. compound (Tissue-Tek Sakura Finetek, Torrance, CA, USA) for subsequent analysis.

The study was approved by the institutions’ Boards of Clinical and Experimental Research and complied with the provisions of the Good Clinical Practice guidelines and the Declaration of Helsinki. All patients provided written informed consent before enrollment.

#### Antibodies and reagents

The polyclonal anti-CNT1 antibody was previously generated and characterized in our laboratory [20]. The CNT1-G7 antibody was purchased from Santa Cruz Biotechnologies (Dallas, TX, USA); the anti-actin antibody was obtained from Sigma-Aldrich (St. Louis, MO, USA); Alexa-488 and Alexa-594 were from Invitrogen (Carlsbad, CA, USA); and anti-mouse and anti-rabbit secondary antibodies were purchased from Bio-Rad (Hercules, CA, USA). Gemcitabine was obtained from MedChem Express (Sollentuna, Sweden), and 5-DFUR was purchased from Sigma-Aldrich.

#### Cell culture

All cell lines used were derived from PDAC and CRC. The PDAC cell lines used, NP9 [21] and CP15T [22], were derived from human pancreatic adenocarcinomas that had been perpetuated as xenograft in nude mice. CRC cell lines were CaCo2 (ATCC, Promochem Partnership, Manassas, VA, USA) and HT29 (ATCC). All cell lines were grown in a monolayer on solid support at 37 °C in a humidified 5% CO_2_ atmosphere. HT29 cells were maintained in Dulbecco's modified Eagle's medium (DMEM; Life Technologies, Carlsbad, CA, USA) supplemented with 10% fetal bovine serum (FBS), 2 mM L-glutamine (Gln) (Life Technologies), 20 U/mL penicillin, and 20 μg/mL streptomycin (Life Technologies). CP15T and NP9 cells were maintained in DMEM and F12 (1:1) (Life Technologies) supplemented with 10% inactivated fetal bovine serum (iFBS), 20 U/mL penicillin, and 20 μg/mL streptomycin (Life Technologies). CaCo2 cells were maintained in MEM (Eagle’s Minimum Essential Medium) (Life Technologies) containing 20% FBS, 2 mM Gln (Life Technologies), 20 U/mL penicillin, and 20 μg/mL streptomycin (Thermo Fisher Scientific). All cell lines were confirmed to be mycoplasma free every 2 weeks by PCR amplification.

#### Spheroid cell culture

Cells (5000 cells/well) were seeded in round-bottom, low-attachment 96-well plates (Corning Cat. 7007; Corning, NY, USA) in a final volume of 100 μl. After centrifuging at 1200 rpm for 7 min without braking, cells were grown for 7 or 10 days depending on the specific experiment. Spheroid growth was analyzed by acquisition of microscopic images daily at 10 × magnification using an AE2000 microscope connected to a Motic Camera 5.0, followed by measurement of area percentage using an ImageJ (Fiji Package) macro adapted from a previous report [23]. Growth was calculated according to the following formula: Percent growth = (area on day 10–area on day 2) × 100. Spheroids from the HT29 cell line were transfected with siRNA, or miRZip vectors on day 5.

#### miRNA silencing and inhibition

miRNA silencing was performed using an antisense approach employing antagomirs (miRCURY LNA microRNA Inhibitors; Exiqon, Vedbaek, Denmark) of selected miRNAs and negative control antagomirs. Cells were transfected with each siRNA (30 nM) using Transit-siQuest (Mirus Bio, Madison, WI, USA), and spheroids were transfected with Lipofectamine 3000 (Life Technologies) according to the manufacturers’ instructions. miR-106a was also knocked down using the shRNA-expressing vector, miRZip-106a (System Bioscience, Palo Alto, CA, USA). The negative control-expressing vector was synthesized by introducing a luciferase-targeting shRNA through restriction digestion and cloning. Cells were transfected with 2 μg of miRZip vector using Lipofectamine 2000 (Life Technologies), and spheroids were transfected with 0.1 μg of miRZip vector using Lipofectamine 3000 (Life Technologies) according to the manufacturer’s instructions.

#### RNA isolation and RT-PCR

Total RNA was isolated from cell lines, spheroids, and tumors using an miRNeasy Mini kit (Qiagen, Venlo, Netherlands) according to the manufacturer’s instructions. Total RNA (1 μg) was reverse transcribed into cDNA using M-MLV Reverse Transcriptase (Invitrogen) and random hexamers (Invitrogen). A total of 8 ng of RNA was reverse transcribed to cDNA for miRNA analysis using TaqMan Advanced miRNA Assays (Life Technologies). Analyses of hCNT1, hCNT2, hCNT3, hENT1, hENT2 and GAPDH (endogenous control) mRNA and miRNA levels were determined by RT-PCR using TaqMan Gene Expression Assays (Applied Biosystems, Foster City, CA, USA) as previously described [24]. Primers and probes used to amplify miRNAs in real-time PCR were purchased from Applied Biosystems (Life Technologies). Relative gene expression was quantified using the ΔΔCT method as described in the TaqMan user’s manual (User Bulletin no. 2; Applied Biosystems). Gene expression levels for nucleoside transporters were normalized to that of the GAPDH gene, whereas miRNA levels were normalized to that of *SNORD48*. The amount of mRNA is expressed in arbitrary units (AU).

#### Protein isolation and Western blot analysis

Whole-cell extracts were obtained by lysing cells with lysis buffer (20 mM Tris–HCl pH 8.0, 150 mM NaCl, 10 mM EDTA, 10 mM Na_4_P_2_O_7_, 2 mM Na_3_VO_4_, 100 mM NaF, 1 mM β-glycerophosphate, 1% Igepal CA-630) containing 1% Complete Mini protease inhibitors (Roche, Mannheim, Germany) and 1% phosphatase inhibitors cocktail (PhosSTOP, Roche). Protein concentration in lysates was determined by Bradford assay (Bio-Rad), and equal amounts of protein (80–100 μg) from each sample were resolved by sodium dodecyl sulfate–polyacrylamide gel electrophoresis (SDS-PAGE) on 10% gels and transferred to polyvinylidene difluoride (PVDF) membranes using standard methods. Membranes were immunoblotted with the indicated primary antibodies, and immunoreactive proteins were detected using a chemiluminescence detection kit (Bio-Rad).

#### Immunofluorescence analysis

Clinical samples embedded in O.C.T. were cut into 10-μm sections using a cryostat and slide mounted. The slides were then fixed by incubating with 4% paraformaldehyde (PFA) at room temperature for 1 min. Spheroids were fixed by incubating with 4% PFA at room temperature for 30 min. Samples were rinsed three times with phosphate-buffered saline (PBS), subsequently permeabilized with 0.05% Tween-20 in PBS for 30 min (15 min with 0.2% saponin in the case of tissue slides), and rinsed again three times with PBS. Clinical samples and spheroids were incubated overnight with polyclonal anti-CNT1 and monoclonal anti-CNT1 antibodies, respectively, followed by incubation with secondary antibodies for 4 h. After costaining with bisbenzimide H 33342 1 μg/mL (Sigma-Aldrich) to label nuclei, samples were mounted with ProLong Gold antifade reagent (Life Technologies). Images were obtained with a laser-scanning confocal microscope (Leica SPE, Leica Microsystems) and analyzed using ImageJ software (Fiji Package).

#### *3*′*-UTR luciferase vector construction and site-directed mutagenesis*

The 3′-UTR of hCNT1 was amplified from HT29 genomic DNA using Phusion High-Fidelity DNA polymerase (Life Technologies) and cloned to the end of a luciferase reporter gene into the pGL3-promoter vector using the primers, 5′-CTA GTC TAG AGG ACA GAA CAT GCT TGT GC-3′ (forward) and 5′-CTA GTC TAG ATA AAC AGC CCT CTC TAA G-3′ (reverse). Binding sites (BS) were deleted by directed site-mutagenesis using Phusion High-Fidelity DNA polymerase and the following phosphorylated (PNK, NEB; Ipswich, MA, USA) primer pairs: miR-17/106a BS, 5′-CTG AGG GCT GTT CTC CCC CGG GAA C-3′ (forwards) and 5′-TGT TCT GTC CTC TAG AAT TAC ACG GCG A -3′ (reverse); and miR-18a BS, 5′-CCC TTT CCC AGA GCC-3′ (forward) and 5′-GGG ACA GAT GGT TCC-3′ (reverse). All constructs were verified by DNA sequencing (BigDye Terminator v3.1; Applied Biosystems).

#### *Luciferase 3*′*UTR-reporter assay*

Cells were seeded into 24-well plates. After 24 h, cells were co-transfected (total DNA, 750 ng/well) with either WT or mutant 3′-UTR–expressing vectors and a pRL-TK vector using Lipofectamine 2000 (Life Technologies). Forty-eight hours after transfection, luciferase assays were performed using a Dual-Luciferase Reporter Assay System (Promega, Madison, WI, USA). The 3′UTR sequence being targeted by miRNA will decrease luciferase activity.

#### Cell viability assay

Spheroids grown as indicated above were treated on day 7 with 20 nM gemcitabine or 50 nM 5-DFUR. Cell viability assays were performed on day 10 using CellTiter-Glo 3D (Promega, Cat. G9683) as described by the manufacturer. Briefly, the reagent CTG was added to each well (10% of well volume), and 100 μl of lysed cells was transferred to white plates, after which luminescence was read using a Glomax® 96 Microplate Luminometer (Promega). Luminescence is recorded as relative light units (RLU) and expressed as percent luminescence.

#### Transport assay

Cytidine uptake rate was measured by incubating spheroids at room temperature for 10 min with [^3^H]-labeled cytidine (1 μM, 1 μCi/mL; Moravek Inc., Brea, CA, USA) in sodium-rich transport medium (137 mM NaCl, 5 mM KCl, 2 mM CaCl_2_, 1 mM MgSO_4_, 10 mM HEPES, pH 7.4) containing dipyridamole 10 μM (Sigma-Aldrich) to block equilibrative transport. Transport was stopped by washing with cold stop solution (137 mM NaCl, 10 mM HEPES pH 7.4). Cells were solubilized with 100 mM NaOH containing 0.5% Triton X-100. Protein concentration was determined using a bicinchoninic acid (BCA) assay (Pierce, Life Technologies), and the remaining volume was used for counting radioactivity.

#### Statistical analysis

Correlations between hCNT1 and microRNAs were performed using GraphPad Prism (La Jolla, CA, USA) and analyzed by one-way analysis of variance (ANOVA) using a post hoc Tukey test. Clinical samples and results from in vitro assays were analyzed using unpaired Student’s *t* test. In all cases, differences were considered significant at *p *values < 0.05.

## Results

### Loss of hCNT1 in CRC and PDAC

Despite the key role played by concentrative nucleoside transporters in nucleotide salvage and uptake of nucleoside-derived drugs, baseline expression of these transporters in healthy tissues in the human body has not been comprehensively established. To address this, we explored the publicly available databases E-MTAB-2836 and E-PROT-29 for mRNA and protein expression, respectively [25, 26]. In general, expression of hCNT family members in human tissues is more restricted than that of their equilibrative counterpart, hENT1 (Supplementary Fig. 1). Among hCNTs, hCNT1 protein was mainly detected in placenta, kidney and the digestive system (Supplementary Fig. 1), particularly in differentiated cells [27]. Considering its ability to transport nucleoside-derived drugs, its role beyond mere substrate-translocation functions and its decreased expression in some tumors, we analyzed hCNT1 expression in two different digestive cancers currently treated with fluoropyrimidines: colorectal carcinoma (CRC) and pancreatic ductal adenocarcinoma (PDAC). This analysis revealed a significant decrease in hCNT1 mRNA levels in PDAC clinical samples compared with that in healthy pancreatic samples, confirming previously published results [17]. In contrast, no differences in hCNT1 mRNA expression were observed in CRC (Fig. 1A).

Given the lack of information regarding hCNT1 protein expression in either type of cancer, we next assessed levels of this protein in CRC and PDAC samples and matching non-tumoral adjacent tissues using immunofluorescence. hCNT1 protein was detected in the luminal epithelium and in crypt glands in colonic samples, and in intercalated and larger ducts in pancreatic tissues. Unlike mRNA levels, hCNT1 protein levels showed a clear decrease in both cancers (Fig. 1B), although the decrease in PDAC was significant only in intercalated ducts (Supplementary Fig. 2).

**Fig. 2:**
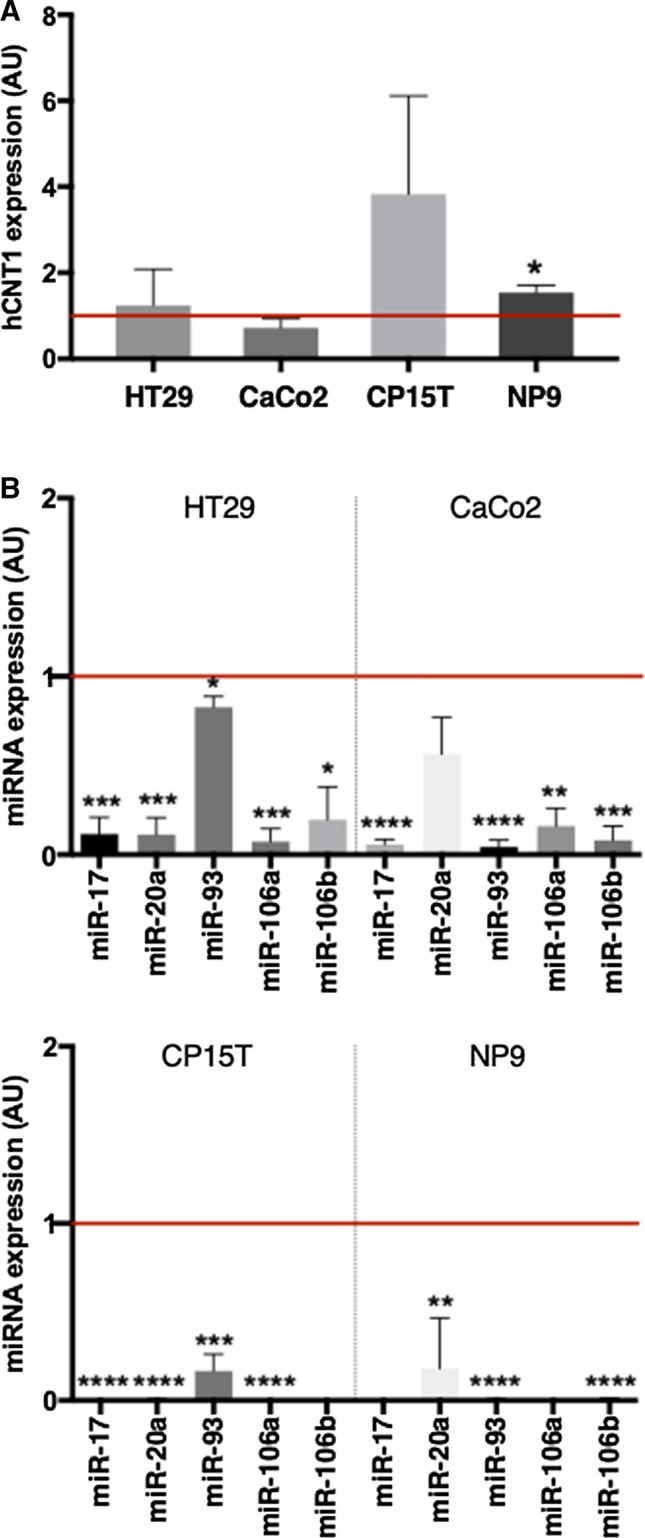
Modulation of hCNT1 by miR-17 family silencing. hCNT1 mRNA expression after knocking down miR-17 family members for 48 h in the indicated CRC and PDAC cell lines. **B** Individual miRNA expression following miR-17 family knockdown in each cell line. The corresponding control, in arbitrary units (AU), is indicated with a horizontal line. All RNA results are expressed as means ± SEM (*n* = 3) and are presented as fold change (*p < 0.05, **p < 0.01, ***p < 0.005, ****p < 0.001; unpaired Student’s *t* test)

To ascertain the possible cause of the diminished expression of hCNT1 in cancer, we looked for the presence of mutations and alterations in methylation in the *SLC28A1* gene by analyzing data from the TCGA-COAD (colon adenocarcinoma) and TCGA-PAAD (pancreatic adenocarcinoma) projects. This analysis of *SLC28A1* mutation status showed the presence of simple somatic mutations in 3.50% and 1.10% of CRC and PDAC cases, respectively. Copy number variation (CNV) events were also found in only a small percentage of cases (< 5%). In addition, an analysis of data from the same projects using TCGA Wanderer software [28] showed no increases in methylation. Given the low frequencies of somatic mutations and CNVs and the absence of methylation changes, we extended our investigation into possible causes of diminished hCNT1 expression in cancer by analyzing putative miRNA binding sites in the 3′-UTR of hCNT1 mRNA. To this end, we analyzed the miRWalk database [29] for candidate miRNAs that were increased in CRC and/or PDAC using RNAHybrid [30], PITA [31] and miRmap [32]; the canonical hCNT1 (NM_0012287762.2/NM_ 004,213.5) was used as a reference sequence. Among identified miRNA candidates were five members of the miR-17–92 cluster family: miR-17, miR-20a, miR-106a, miR-106b and miR-18a (Supplementary Fig. 3). The well-established relationship between members of the miR-17 family and several kinds of cancers prompted us to silence all family members in CRC- and PDAC-derived cell lines (Fig. 2B). Simultaneous knockdown of these miRNA family members induced a significant increase in hCNT1 mRNA levels in the PDAC cell line NP9 (Fig. 2A). No significant changes were observed in CRC cell lines (Fig. 2A).

### hCNT1 mRNA is a direct target of miR-17 and miR-106a

**Fig. 3:**
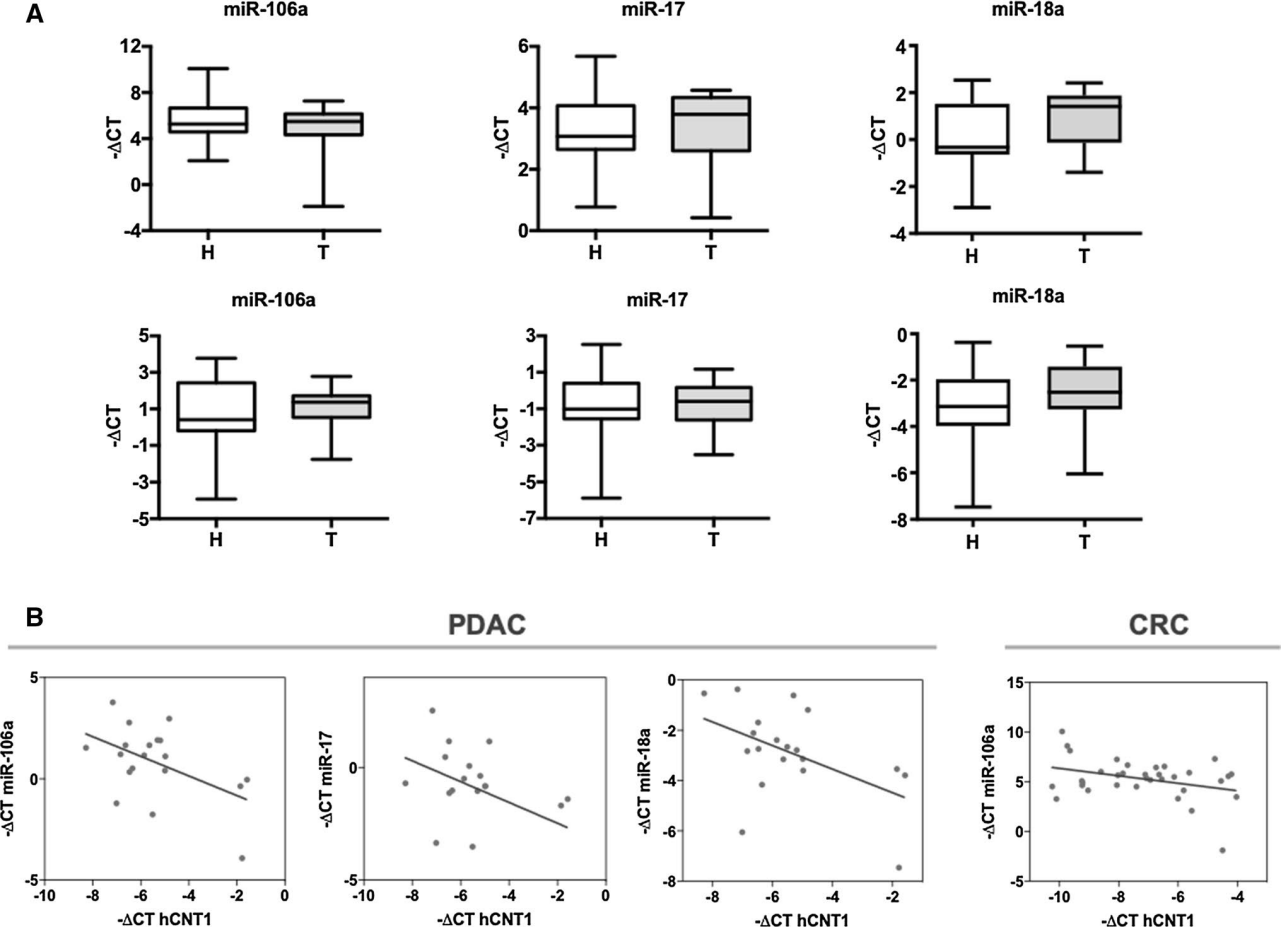
Aberrant expression of miRNAs is correlated with hCNT1 loss in CRC and PDAC. **A** miR-106a, miR-17 and miR-18a levels in clinical samples of CRC (*n* = 17; top) and PDAC (*n* = 10; bottom) tumor and normal adjacent tissues. Statistical significance was determined using unpaired Student’s *t *test. **B** Significant negative correlations between miRNA candidates and hCNT1 in CRC and PDAC. *R* = 0.34 in CRC; *R* = 0.5, *R* = 0.44 and *R*2 = 0.48 in PDAC for miR-106a, miR-17 and miR-18a, respectively. Statistical significance was determined by one-way ANOVA (*p* < 0.05)

Focusing on those miRNAs most relevant to hCNT1 regulation in a clinical setting, we next measured the expression of miRNA candidates in clinical CRC and PDAC samples (Fig. 3A). A correlation analysis of miRNAs and hCNT1 mRNA showed a significant negative correlation between hCNT1 and miR-106a in CRC and PDAC, and with both miR-17 and miR-18a in PDAC only (Fig. 3B). hCNT1 mRNA levels were similarly decreased in hepatocarcinoma (HCC), where it also showed a significant negative correlation with miR-106a and miR-18a levels (Supplementary Fig. 4).

**Fig. 4:**
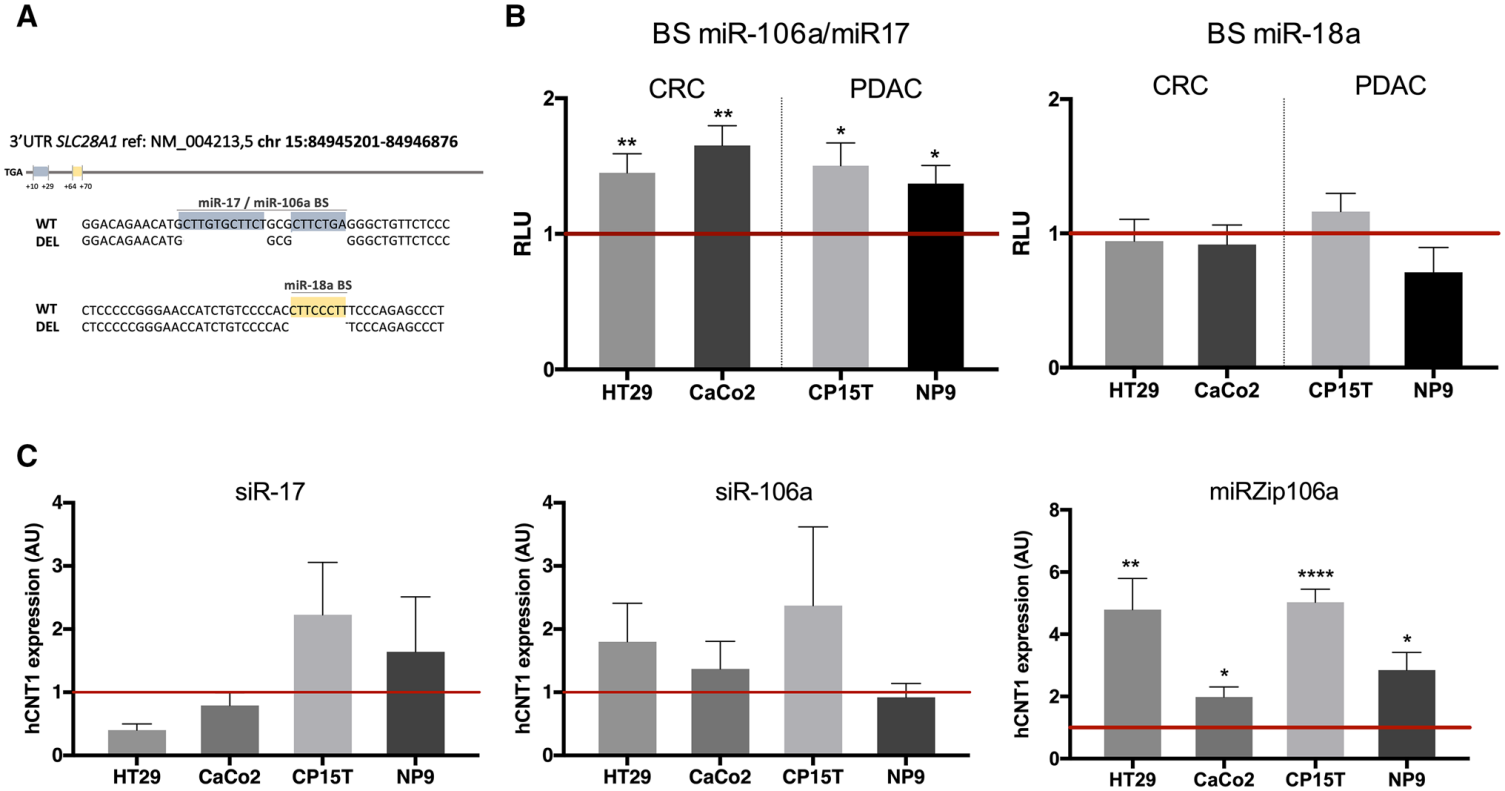
hCNT1 is a direct target of miR-106a and miR-17. **A** Schematic representation of hCNT1 3′UTR, with the relative positions of miRNAs binding site (BS) included (up). Detail of deleted sequences corresponding to miR-106a/miR17 BS (from + 10 to + 29 relative to TGA) and miR-18a (from + 64 to + 70) (down). **B** Validation of miRNAs binding to hCNT1 3′UTR by luciferase assay. Cells were transfected with wild type 3′UTR (WT) or modified 3′UTR (MUT), and pRL-TK (renilla) vector as transfection control, and 48 h later luciferase assay was performed. Luciferase activity of 3′UTR MUT was normalized to WT (represented as 1, horizontal line). Values are represented as mean ± SEM (*n* = 3). Statistical significance was determined with Student’s *t* test. **C** Changes in hCNT1 expression caused by specific miRNA modulation. Cells were transfected for 48 h with either the indicated siRNAs or miRZip (shRNA) vectors. hCNT1 mRNA expression in arbitrary units (AU) is presented as fold change compared with each control, depicted as a horizontal line. Values are presented as means ± SEM (*n* = 3; *p < 0.05, **p < 0.01, ****p < 0.001; unpaired Student’s *t* test)

Considering that miR-17 and miR-106a share the same binding site in the 3′-UTR of *SLC28A1* that is distinct from that of miR-18a, we separately deleted the two binding sites in the 3′-UTR. CRC and PDAC cell lines were transfected with the different 3′-UTR expressing vectors and miRNA specific binding was assessed using a luciferase-based reporter assay (Fig. 4A). Deletion of the shared miR-106a/miR-17 binding site induced a significant increase in luciferase activity in all cell lines, whereas deletion of the miR-18a binding site had no effect (Fig. 4B). Changes of hCNT1 mRNA expression showed a trend toward an increase after silencing miR-106a in both CRC and PDAC cell lines, whereas this trend was only observed in PDAC cell lines following miR-17 silencing (Fig. 4C). These different results in PDAC and CRC are in accord with the observed clinical correlations, in which only miR-106a was significantly associated with both cancers (Fig. 3). Therefore, the role of miR-106a in hCNT1 expression was further analyzed by inhibiting it using a small hairpin RNA (shRNA) against miR-106a (miRZip system). miR-106a inactivation using the shRNA, miRZip-106a, significantly increased hCNT1 mRNA expression in all four cell lines tested (Fig. 4C). Notably, although miRZip-106a is designed to target miR-106a, the high complementarity between miR-106a and miR-17 (Supplementary Fig. 5) could result in miR-17 knockdown, suggesting a possible contribution of miR-17 to the observed strong effect.

### hCNT1 is functionally regulated by miR-106a and miR-17

**Fig. 5:**
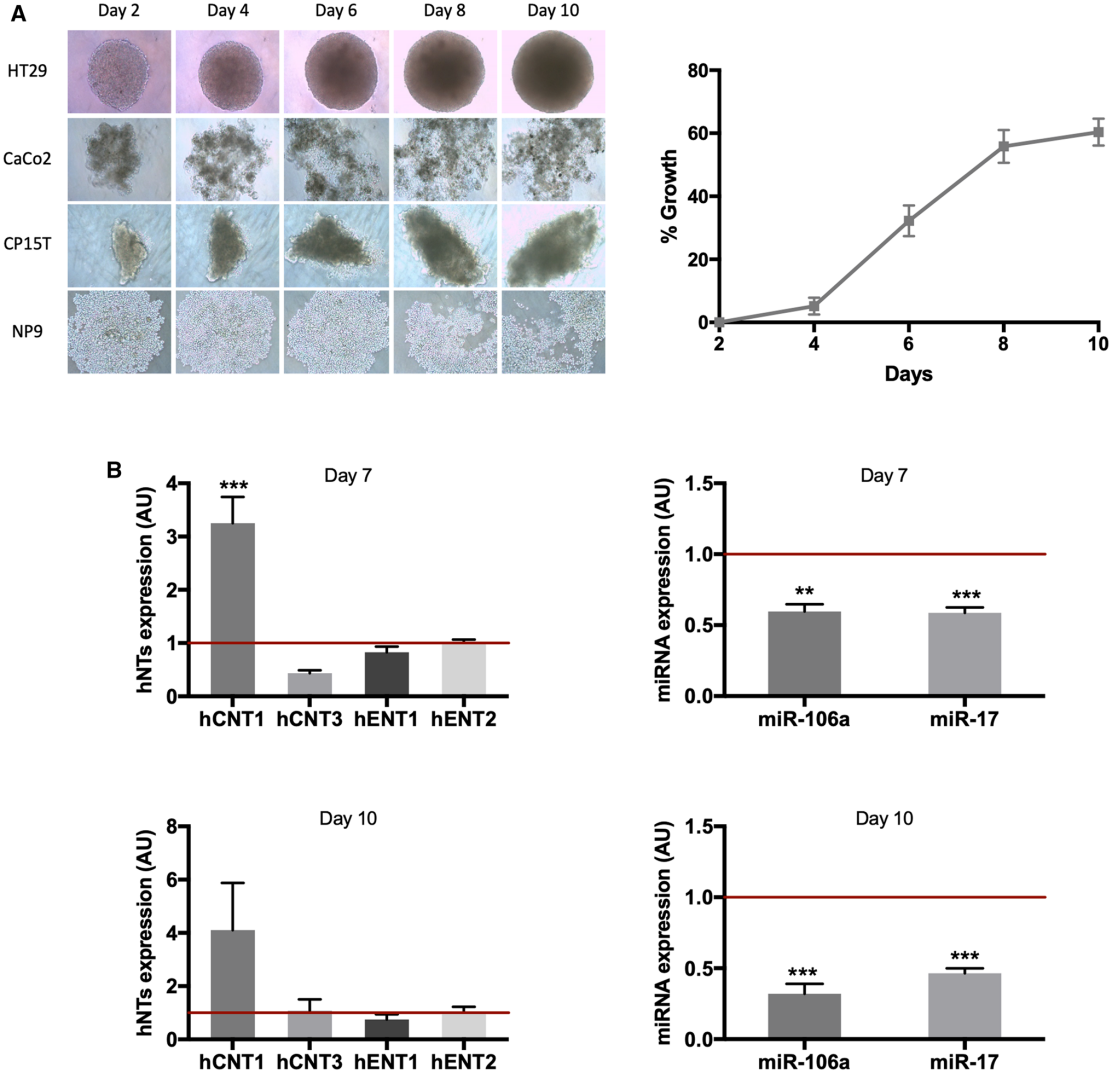
Characterization of the 3D culture spheroid model. **A** Left: Of the tested CRC and PDAC cell lines, only HT29 was able to grow as spheroids. Right: Analysis of HT29 spheroids growth over the course of 10 days. **B** Changes in the expression of nucleoside transporters accompanying formation of a 3D spheroid. Expression of miRNAs and mRNAs for NTs was determined 7 (upper) and 10 (lower) days after initiating spheroid growth and compared with endogenous expression in cells grown in a monolayer model (represented as 1, horizontal line; in arbitrary units [AU]). Results are expressed as means ± SEM (*n* = 3–5; **p < 0.01, ***p < 0.005; unpaired Student’s *t* test)

**Fig. 6:**
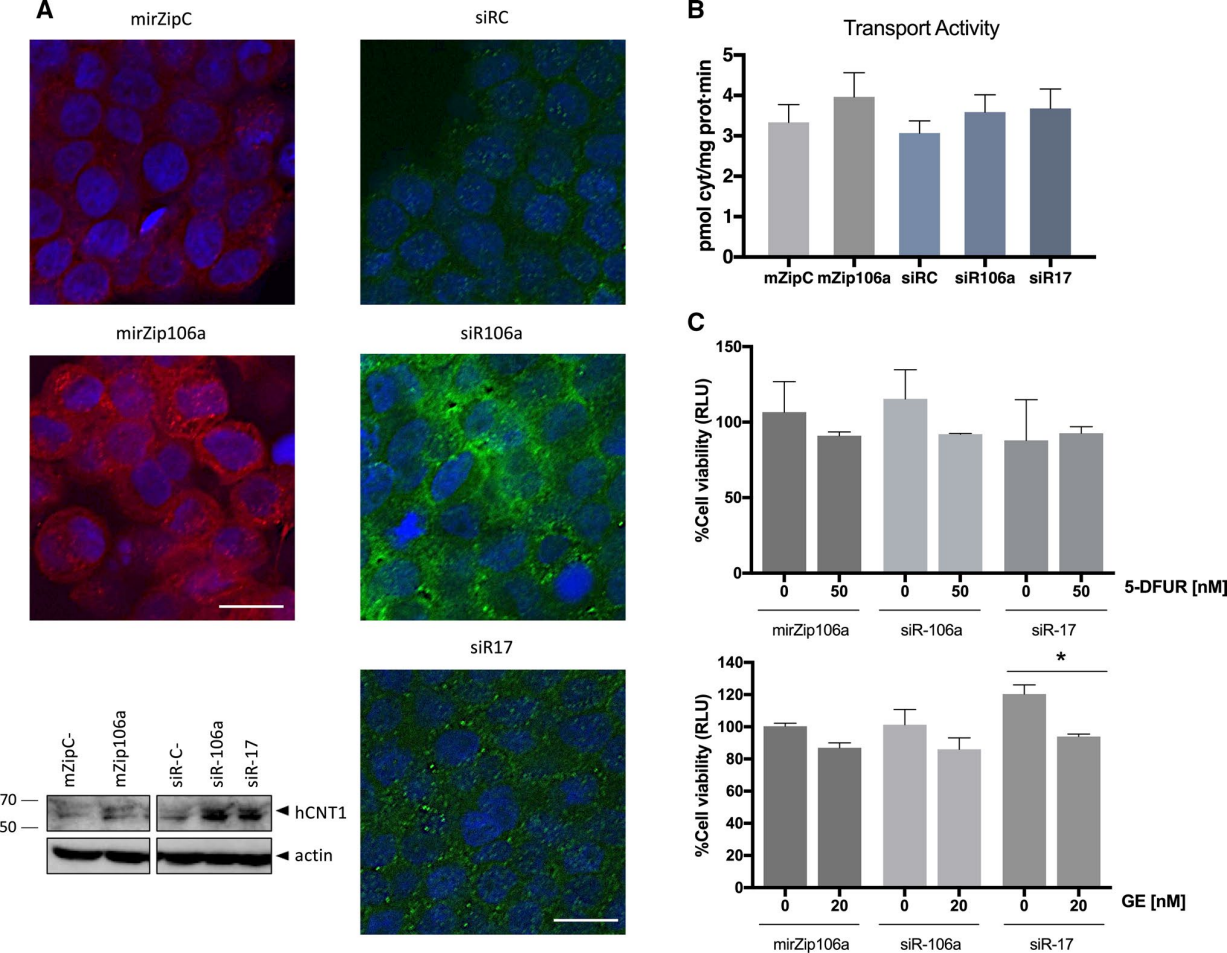
miRNAs modulate expression and activity of hCNT1 in a three-dimensional cell culture model. Spheroids were transfected on day 5 of growth, and protein and activity were determined at 48 h post transfection (day 7 of spheroid growth). **A** Alterations of hCNT1 protein expression induced by modulation of miRNA. Representative experiment showing immunolocalization of hCNT1, determined by confocal microscopy. Nuclei were stained with Hoechst (blue), and hCNT1 protein was detected in red and green for miRZip (shRNA)- and siRNA-transfected spheroids, respectively. All the images were obtained under the same magnification. Scale bars, 10 µm. **A** representative Western Blot is shown. **B** hCNT1-dependent transport of [3H]cytidine (right) was calculated as the difference between cytidine uptake in medium without versus with dipyridamole. All results are expressed as means ± SEM (*n* = 3–5). Statistical significance was determined by unpaired Student’s *t* test. **C** Spheroid viability was measured at day 10 of spheroid growth after combined miRNA modulation and 5-DFUR (up) or gemcitabine (down) treatment. Spheroids were transfected at day 5 of growth and treated with 50 nM 5-DFUR or 20 nM gemcitabine for 72 h beginning at day 7. Cell viability was analyzed as a percentage of detected relative light units (RLU) compared with its corresponding transfection control. All values are presented as means ± SEM (*n* = 3; *p < 0.05; Student's *t* test)

The difficulties in detecting hCNT1 because of its low endogenous expression prevented performing miRNA gain-of-function assays, prompting us to find a better model to further study miRNA-related hCNT1 regulation. Indeed, hCNT1 expression under physiological conditions appears to be restricted to differentiated cells; even in these cells, little or no expression is detected in most cases. Thus, we sought to grow the same cell lines as multicellular spheroids under non-adherent conditions to improve cell–cell and cell–matrix interactions. Only HT29 cells formed aggregated spheroids under our culture conditions, and showed exponential growth between days 4 and 8 after initiation (Fig. 5A). An expression analysis of nucleoside transporters revealed an increase in hCNT1 mRNA levels in HT29-derived spheroids compared with that in monolayer cultures, but showed no significant changes in hCNT3, hENT1 or hENT2 mRNA levels. Concomitant with this, miR-106a and miR-17 levels significantly decreased (Fig. 5B). siRNA-mediated knockdown of miR-17 and miR-106a induced an increase in hCNT1 protein, as measured by Western blotting and immunofluorescence (Fig. 6A). More consistent results were obtained in HT29-derived spheroids using siRNA than using shRNA (miRZip), possibly owing to limitations of transferring miRZip into spheroids because of the size of the vector compared with that of the siRNA.

To confirm that hCNT1 was properly and functionally located at the cell membrane in spheroids, we assessed cytidine uptake using the previously described methods to silence miR-106a and miR-17. Under conditions of miR-106a and miR-17 knockdown using both approaches, nucleoside uptake rates in spheroids showed an increasing trend, although this difference fell short of statistical significance (Fig. 6B). In this context, it is important to note that the observed transport rates probably only reflect nucleoside uptake at the spheroid surface; thus, our experimental conditions would tend to underestimate increases in nucleoside uptake.

Since hCNT1 can efficiently transport nucleoside-derived drugs, such as gemcitabine and 5-DFUR (a metabolite of capecitabine and the precursor of 5-fluorouracil), we sought to determine whether modulation of miRNAs might impact the cytotoxicity of these drugs by increasing their bioavailability. Spheroids were treated with drugs for 72 h on day 7 of spheroid growth, a time when hCNT1 expression had already increased. 5-DFUR (50 nM) treatment of spheroids formed from HT29 cells in which miR-106a or miR-17 had been knocked down did not significantly alter cell viability, although a trend toward a decrease was observed with siRNA-mediated miR-106a knockdown. Nevertheless, 20 nM gemcitabine treatment was significantly more efficient after miR-17 silencing and showed an increasing trend with miR-106a knockdown (Fig. 6C).

## Discussion

The requirement of cancer cells for abnormally high levels of nucleotides to support their high rates of DNA synthesis and cell proliferation would predict upregulation of NT expression so as to fuel nucleoside salvage pathways. However, the actual situation appears to be more complex and, at least for hCNT1, even opposite that of expectations. Indeed, expression analyses have shown downregulation of hCNT1 at the mRNA level in HCC, PDAC, and cholangiocarcinoma tumors [15, 17–19, 33]. However, these analyses, performed using homogenized samples that included the multiple cell types present in tumors—and even normal tissues—could underestimate hCNT1 levels. Indeed, analyses of hCNT1 mRNA and protein levels in 29 healthy tissues [25, 26] showed differences in the detection of mRNA and protein, with twice as much protein as mRNA being detected in the studied tissues. In fact, hCNT1 immunohistological analyses have only been performed on gynecologic and breast tumors, in both cases showing a decrease in protein expression [14, 16]. In this context, our immunohistological analysis showed a clear decrease in hCNT1 protein in CRC and PDAC, likely owing to its restricted expression in specific differentiated cells. Moreover, the only published study of hCNT1 expression and localization in human small intestine demonstrated selective expression in enterocytes, with higher expression in the upper part of villi [27] than in crypts. Although loss of hCNT1 appears to be a common event in several kinds of cancers, its cause is still uncertain and little is known about how hCNT1 expression is regulated. One study on pancreatic cancer cells performed using the gemcitabine-resistant cell line, MIA PaCa-2, suggested modulation of hCNT1 expression by proteasomal inhibitors or selective miRNA antagonists [17]. The four identified miRNAs (miRNA-122, miRNA-214, miRNA-339–3p, and miRNA-650) were able to significantly reduce hCNT1 protein levels. However, the lack of a specific interaction analysis of these miRNAs with the 3′-UTR of hCNT1 does not allow to confirm a direct effect on hCNT1.

miRNAs are involved in cancer pathogenesis and can exhibit different abnormal expression signatures that characterize diverse types of cancers. One of the best studied is the miR-17–92 polycistronic cluster, whose members are used for subtype profiling of a variety of cancers [34]. This cluster is highly conserved in mammals and has two paralog clusters: miR-106a_363 and miR-106b_25 [35]. Alteration of members of the three clusters has been demonstrated in CRC and PDAC [36, 37].

In this work, among the putative candidates, only miR-106a and miR-17, which share the same seed sequence, were validated as being able to modulate the expression of the transporter protein in monolayers and in spheroid cultures. hCNT1 is mainly expressed in polarized epithelia, where its variable expression along the intestine is a unique feature of differentiated enterocytes [38]. With the goal of finding a better model for studying the modulation of hCNT1, we chose to work with spheroids because they mimic a polarized structure and show improved cell–cell interactions [39]. Our results showed that basal hCNT1 expression in HT29 spheroids increased in the absence of significant changes in other NTs involved in the uptake of nucleoside analog drugs used in current treatments of solid tumors.

CRC and PDAC are complex and heterogeneous diseases treated with chemotherapy regimens primarily based on fluoropyrimidines, such as 5-fluorouracil (5-FU), capecitabine, and gemcitabine (GE) [40, 41]. Capecitabine is an orally administered fluoropyrimidine that is metabolized to 5-FU through a three-step process. The active form, 5-FU, is obtained by thymidine phosphorylase metabolization of 5-DFUR, mostly inside the tumor [42]. The hCNT1 transporter mediates the uptake of both pyrimidine analogs, gemcitabine and 5-DFUR [20, 43]. NTs are the limiting step in allowing nucleoside analog drugs to enter tumor cells, an attribute that explains their widely demonstrated role in chemoresistance [38]. Accumulating evidence suggests that epigenetic alterations, including dysregulation of miRNAs, are contributors to drug resistance [44, 45]. Chemoresistance is mediated by various mechanisms, including aberrant metabolism, alterations in ATP-binding cassette (ABC) transporter activity and resistance to apoptosis, among many others, and it has been reported that miRNA alterations can contribute to all of these mechanisms [46]. Collectively, our results obtained in spheroids, including data on hCNT1 expression and transport activity as well as cell viability after 5-DFUR or gemcitabine treatment, point to a contribution of hCNT1 to chemoresistance, reflecting miR-106a- and miR-17-mediated actions on the transporter transcript. Gemcitabine results were more consistent in this respect, likely because the affinity of gemcitabine for hCNT1 is about an order of magnitude higher than that for hENT1, whereas the difference in affinity for 5-DFUR is only about twofold [20, 43, 47]. Furthermore, differences in the mechanisms of action and enzymes responsible for metabolizing both drugs cannot be dismissed, given that all proteins involved in these processes can simultaneously be targeted by miR-106a and miR-17, which thereby contribute to treatment outcome in different ways.

Several studies have found a correlation between the levels of these miRNAs and chemoresistance to these pyrimidine analog drugs. miR-17 expression has been associated with a worse prognosis in CRC treated primarily with fluorouracil-based chemotherapy regimens combined with leucovorin and oxaliplatin [48]. A six-miRNA signature that includes miR-17 was shown to predict treatment responses of metastatic CRC to first-line systemic treatment regimens containing 5-FU or capecitabine [49]. Plasma miR-17–92 cluster level was associated with the progression of advanced gastric cancer and effectiveness of capecitabine chemotherapy [50]. High levels of miR-106a in the serum of non-small cell lung cancer (NSCLC) patients can be useful in establishing non-responder patients during gemcitabine and cisplatin chemotherapy [51]. Although the effects of these two miRNAs (miR-106a and miR-17) on hCNT1 may not be the key factor that induces chemoresistance, it could certainly contribute to hindering the tumor bioavailability of these drugs. Understanding the complex mechanisms underlying chemoresistance in PDAC and CRC is essential for optimizing current therapeutic strategies and rational approaches to developing new treatments.

In addition to their utility as biomarkers of response to therapy, miRNAs have received increasing interest as biomarkers for the diagnosis and prognosis of many kinds of cancer. In this sense, miR-17 and miR-106a have been recognized as significant diagnostic and/or prognostic biomarkers in CRC [52, 53], PDAC [54], gastric cancer [55], and NSCLC [51]. Importantly, these miRNAs can also be detected in biological fluids, which can easily be collected at different time points, making these mRNAs even more relevant as non-invasive biomarkers for monitoring disease progression and chemotherapeutic response [56, 57]. It has also been found that miR-106a and miR-17 levels are increased in circulating exosomes in pancreatic cancer precursor lesions [58, 59].

Oncogenic miRNAs underlie epigenetic alterations that are undeniably recognized as cancer hallmarks. The current study establishes for the first time a correlation between the oncomiRs, miR-106a and miR-17, and the apparently widespread event of hCNT1 loss in carcinomas. The impact of this regulation on chemoresistance cannot be dismissed, especially considering that hCNT1 is a high-affinity transporter for many of the fluoropyrimidine drugs currently used in cancer treatment.

## Conclusions

Expression of the high-affinity nucleoside transporter hCNT1 is decreased by the commonly deregulated oncomiRs miR-106a and miR-17 which could contribute to chemoresistance to fluoropyrimidine-based treatments.

## Supplementary Information

Below is the link to the electronic supplementary material.

Supplementary file1 (DOCX 6673 KB)

## Data Availability

All data generated or analyzed during this study are included in this published article and are available from the corresponding author on reasonable request. Public data analyzed that support the findings of this study are available at the EMBL-EBI repository (E-MTAB-2836 and E-PROT-29) and at GDC data portal (TCGA-COAD and TCGA-PAAD).
