# Supplementary material for: OncomiRs miR-106a and miR-17 negatively regulate the nucleoside-derived drug transporter hCNT1
Source: Cell Mol Life Sci. 2021 Oct 13;78(23):7505–18. doi: 10.1007/s00018-021-03959-8 (PMC8629896; doi:10.1007/s00018-021-03959-8)
Supplement: Supplementary file 1 — Supplementary file1 (DOCX 6673 KB) [file 18_2021_3959_MOESM1_ESM.docx]

**Supplementary material**


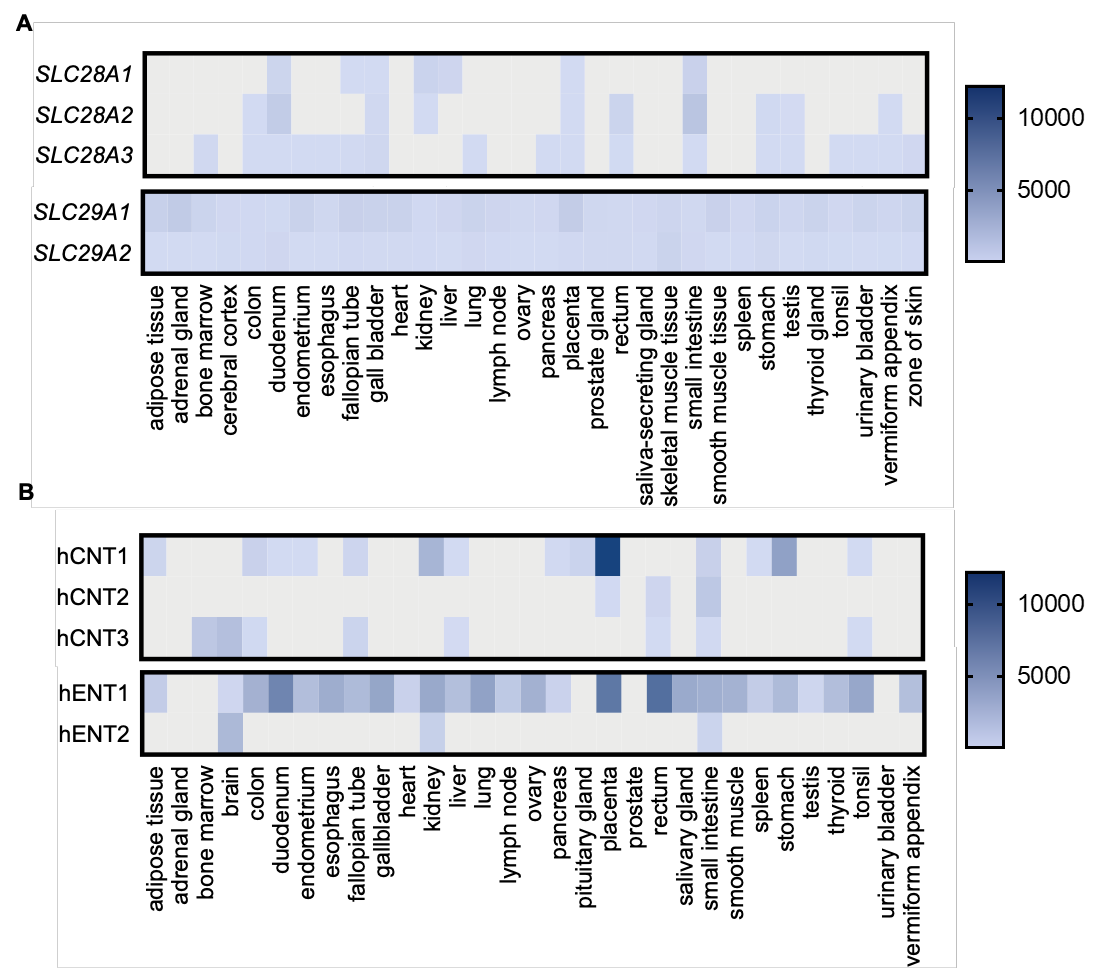


**Supplementary Figure 1. Nucleoside transporters expression in human tissue.** Expression distribution of *SLC28A1*, *SLC28A2*, *SLC28A3, SLC29A1* and *SLC29A2* at RNA **(A)** and protein **(B)** levels. Data obtained from EMBL-EBI database.

**Supplementary Figure 2. hCNTs expression in nontumoural and tumour tissue.** hCNT1 immunofluorescence quantification in pancreatic tissue. Fluorescence was measured in lumen (left) and duct (right) of the indicated samples. Statistical significance was determined with Student’s t-test; p<0,005***.

**Supplementary Figure 3**. **miRNAs related to hCNT1 expression**. Schematic representation **(A)**and corresponding sequences**(B)**of the three paralogous clusters miR-17-92, miR-106a-363, and miR-106b-25. **(C)**miRNA classification within the miRNA families that are part of these clusters.

**Supplementary Figure 4. Aberrant expression of miRNAs correlates with hCNT1 loss in hepatocarcinoma (HCC).** Analysis of hCNT1 mRNA expression **(A)** and miRNAs candidates expression **(B)** in paired clinical samples of tumour (T) and non-tumour adjacent tissues (NT) of HCC (n=22). Statistical significance was determined by Student’s t-test; p<0.05*, p<0.005***, p<0.001****. **(C)** Negative significant correlations between hCNT1 and miR-106a and miR-18a. Statistical significance was determined by 1-way ANOVA (p<0.05).


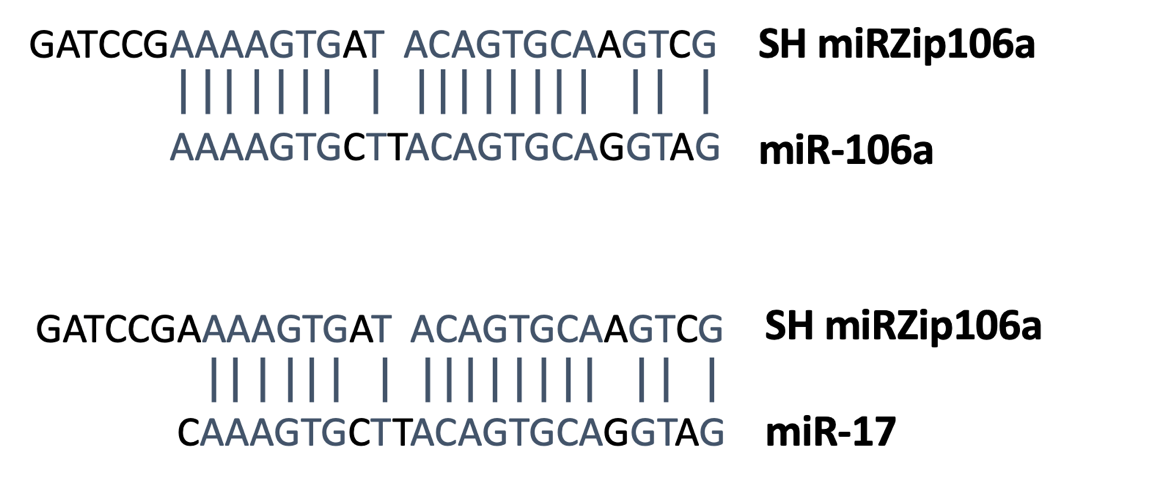


**Supplementary Figure 5. Schematic representation of Sh-miRZip106a sequence and its complementarity to miR-106a and miR-17.**

**Supplementary Table 1. Patient and tumour samples characteristics, indicating the number of samples in each category.** s.d, standard deviation. HCC differentiation degree according to AJCC 8th edition.G1: well differentiated; G2:moderately differentiated; G3: poorly differentiated; G4: undifferentiated. HCV: Hepatitis C Virus infection; NASH: Non Alcoholic Fatty Liver Disease; ALD: Alcohol Liver Disease
